# Identification of Novel Genes Mediating Survival of *Salmonella* on Low-Moisture Foods via Transposon Sequencing Analysis

**DOI:** 10.3389/fmicb.2020.00726

**Published:** 2020-05-15

**Authors:** Victor Jayeola, Michael McClelland, Steffen Porwollik, Weiping Chu, Jeffrey Farber, Sophia Kathariou

**Affiliations:** ^1^Department of Plant and Microbial Biology, North Carolina State University, Raleigh, NC, United States; ^2^Department of Microbiology and Molecular Genetics, University of California, Irvine, Irvine, CA, United States; ^3^Department of Food Science, University of Guelph, Guelph, ON, Canada; ^4^Department of Food, Bioprocessing and Nutrition Sciences, North Carolina State University, Raleigh, NC, United States

**Keywords:** *Salmonella*, mutants, survival, low-moisture foods, desiccation, transposon-sequencing

## Abstract

*Salmonella enterica* is the leading foodborne pathogen associated with outbreaks involving low-moisture foods (LMFs). However, the genes involved in *Salmonella’*s long-term survival on LMFs remain poorly characterized. In this study, in-shell pistachios were inoculated with Tn*5-*based mutant libraries of *S*. Enteritidis P125109, *S*. Typhimurium 14028s, and *S*. Newport C4.2 at approximate 10^8^ CFU/g and stored at 25°C. Transposon sequencing analysis (Tn-seq) was then employed to determine the relative abundance of each Tn*5* insertion site immediately after inoculation (T_0_), after drying (T_1_), and at 120 days (T_120_). In *S*. Enteritidis, *S*. Typhimurium, and *S*. Newport mutant libraries, the relative abundance of 51, 80, and 101 Tn*5* insertion sites, respectively, was significantly lower at T_1_ compared to T_0_, while in libraries of *S*. Enteritidis and *S*. Typhimurium the relative abundance of 42 and 68 Tn*5* insertion sites, respectively, was significantly lower at T_120_ compared to T_1_. Tn*5* insertion sites with reduced relative abundance in this competition assay were localized in DNA repair, lipopolysaccharide biosynthesis and stringent response genes. Twelve genes among those under strong negative selection in the competition assay were selected for further study. Whole gene deletion mutants in ten of these genes, *sspA*, *barA*, *uvrB*, *damX*, *rfbD*, *uvrY*, *lrhA*, *yifE, rbsR*, and *ompR*, were impaired for individual survival on pistachios. The findings highlight the value of combined mutagenesis and sequencing to identify novel genes important for the survival of *Salmonella* in low-moisture foods.

## Introduction

Low-moisture foods (LMFs) have low water activity (A_w_), generally below 0.6, and thus are not conducive to microbial growth (Beuchat et al., 2013). However, the ability of certain foodborne pathogens to persist on LMFs for long periods has rendered these commodities important vehicles for outbreaks of foodborne illness (Beuchat et al., 2013; Finn et al., 2013a). LMFs have been associated with numerous outbreaks, having led to 7,315 cases of foodborne illnesses and 63 deaths worldwide between 2007 and 2012 (Farakos and Frank, 2014). In the United States, an estimated 83% of LMF-associated multistate foodborne outbreaks between 2007 and 2018 have involved *Salmonella* (Centers for Disease Control Prevention [CDC], 2019). Examples of extensively investigated foodborne salmonellosis outbreaks involving LMFs include the 2008–2009 peanut butter outbreak that resulted in 714 cases, 171 hospitalizations and 9 deaths (Centers for Disease Control Prevention [CDC], 2009), and a pistachio-associated salmonellosis outbreak in 2016 (Centers for Disease Control Prevention [CDC], 2016).

The food safety concerns of *Salmonella*-contaminated LMFs are accentuated by the fact that most LMFs are considered ready-to-eat foods and have a relatively long shelf life. Moreover, *Salmonella* contaminating LMFs exhibits unusually high tolerance to other stressors including heat and exposure to bile salts and sanitizers (Gruzdev et al., 2011; Smith et al., 2016). Thus, preventive control and inactivation strategies that may be effective against *Salmonella* contaminating high-moisture foods may be less effective on LMFs. Furthermore, salmonellosis outbreaks associated with LMFs can span several months because *Salmonella* can survive and remain infectious on LMFs for long periods of time (Isaacs et al., 2005; Centers for Disease Control Prevention [CDC], 2009). Indeed, several studies have demonstrated *Salmonella*’s capacity to persist in the absence of growth on a diverse array of LMFs (Kimber et al., 2012; Beuchat and Mann, 2014; Hokunan et al., 2016). However, the molecular mechanisms and genetic determinants mediating long-term survival of *Salmonella* on LMFs are still poorly understood.

Findings from previous studies have suggested that the immediate response of *Salmonella* to desiccation involves accumulation of compatible solutes such as glycine betaine and trehalose (Csonka and Hanson, 1991; Li et al., 2012; Finn et al., 2013a). Transcriptome analyses of *Salmonella* under desiccation on abiotic surfaces suggested that long-term survival involved complex metabolic and cellular processes (Gruzdev et al., 2012a; Li et al., 2012; Finn et al., 2013b; Maserati et al., 2017). Processes and traits found to be upregulated under desiccation included potassium influx, the stress-induced sigma factors (*rpoE* and *rpoS*), Fe-S cluster, fatty acid and amino acid metabolism, stress response, and virulence (Gruzdev et al., 2012a; Finn et al., 2013a; Maserati et al., 2017, 2018). In addition, genes involved in propanoate metabolism, the citrate cycle, and lipopolysaccharide biosynthesis were downregated in *Salmonella* on milk chocolate, powdered milk, black pepper, and dried pet foods (Crucello et al., 2019). Formation of thin aggregative fimbriae and cell filamentation were implicated in desiccation tolerance of *Salmonella* (Mattick et al., 2000; White et al., 2006; Maserati et al., 2017). Most of these studies tested desiccation tolerance of *Salmonella* over relatively short periods of time on dry abiotic surfaces such as plastic, stainless steel, and paper, which may not be representative of the mechanisms employed by *Salmonella* to survive on LMFs.

The objective of the current study was to identify genetic determinants required for survival of *Salmonella* on LMFs. Mutant libraries created by random Tn*5* insertion in the genomes of isolates from three *Salmonella* serovars were selected for both short and long-term survival on in-shell pistachios, followed by transposon-sequencing analysis to identify genetic determinants required for survival. The role of a subset of 12 genes identified via this combined mutagenesis and sequencing approach was then directly investigated by assessing the capacity of single-gene deletion (SGD) mutants to survive on pistachios.

## Materials and Methods

### Bacterial Strains and Growth Conditions

*Salmonella* strains used in this study included *Salmonella enterica* sv Typhimurium strain 14028s, isolated from 4-week-old chickens in 1960 (Jarvik et al., 2010); *Salmonella* Enteritidis PT4 strain P125109, a clinical strain associated with a poultry outbreak in the United Kingdom (Toro et al., 2016); and *Salmonella* Newport strain C4.2, isolated from a tomato field in Virginia, United States (de Moraes et al., 2018). Single gene deletion (SGD) mutants of *S.* Typhimurium strain 14028s harbor a kanamycin (SGD-Kan^R^) or chloramphenicol (SGD-Cm^R^) resistance gene, oriented in the antisense and sense direction, respectively, in regard to the deleted gene (Porwollik et al., 2014). The strains were preserved at −80°C in Luria-Bertani broth (LB; Becton, Dickinson & Co. Sparks, MD, United States) containing 20% glycerol (Fisher Scientific, Fair Lawn, NJ, United States). Bacterial cultures were grown in LB or on LB agar containing 1.2% agar (Becton, Dickinson & Co.) at 37°C for 24 h unless indicated otherwise. When necessary, growth media were supplemented with 60 μg/ml kanamycin (LB + Kan; Fisher Scientific) or 20 μg/ml chloramphenicol (LB + Cm, Acros Organics, NJ, United States).

### Construction of Tn*5* Mutant Libraries

The methods for construction of the barcoded Tn*5* libraries were previously described (de Moraes et al., 2017, 2018). In brief, a library of Tn*5* insertion mutants was constructed for each strain with a mini-Tn*5* derivative into which we inserted an N_18_ random barcode using PCR. This derivative was integrated into the genome using the EZ-Tn*5* <T7/KAN-2> promoter insertion kit (Epicentre Biotechnologies, Madison, WI, United States). Mapping of barcoded transposons to specific locations in the genome was performed as described (de Moraes et al., 2017).

### Negative Selection of Mutant Libraries for Survival on Pistachios

Frozen stocks of mutant libraries were thawed on ice and 1 ml was transferred into two separate 30 ml LB + Kan in 50-ml centrifuge tubes (Becton, Dickinson & Co.) and incubated at 37°C with shaking for 12 h. The cultures were washed twice in 5 ml sterile deionized water (dH_2_O), and combined to yield 10 ml of cell suspension, to be used as inoculum. In-shell pistachios were obtained from a commercial source and analyzed for total aerobic counts and *Salmonella* before use. For inoculation, 200 g of pistachios were weighed into sterile Whirl-Pak stomacher bags (5441 ml, Nasco, Fort Atkinson, WI, United States) and inoculated at 4% volume per weight by transferring 8 ml of the cell suspension in a series of four additions of 2 ml into the bag, each addition followed by vigorous shaking by hand for 30 sec. The inoculated pistachios were dried in a Nalgene polypropylene desiccator (Thermo Fisher Scientific, Waltham, MA, United States) containing drierite desiccant (W.A. Hammond Drierite Co. Ltd., Xenia, OH, United States) for 24 h, at which time the A_w_ approximated that of uninoculated nuts. After drying, the nuts were placed into several 50 ml centrifuge tubes (VWR Int., Radnor, PA, United States), capped tightly, and stored at 25°C in the dark.

*Salmonella* from the inoculated pistachios were recovered immediately after inoculation, after drying, and then after various times of storage up to 120 days. In each case, 10 g of nuts (approximate 10 pistachios) were transferred into 20 ml LB + Kan in 532-ml Whirl-Pak bags (Nasco). The bag was vigorously hand-massaged for 1 min and incubated at room temperature for 10 min. For enumerations, 100 μl of the rinsate was serially diluted in dH_2_O and appropriate dilutions were plated on LB + Kan agar plates, followed by incubation at 37°C for 24 h. The remainder of the mixtures of pistachios and LB + Kan was incubated statically at 37°C for 12 h to amplify the pistachio-associated *Salmonella* populations, shaken manually for 30 s, and 1 ml of the resulting suspension was transferred into a 1.5-ml microcentrifuge tube (Fisher Scientific) and centrifuged at 15,000 × *g*, 25°C for 2 min. The pellets were then preserved at −80°C. The population of *Salmonella* in the enriched mixture was enumerated as described. The experiment was conducted in three independent trials with duplicate samples processed per trial.

### Library DNA Preparation, Sequencing, and Data Analysis

The methods for library DNA preparation, sequencing and data analysis were previously described (de Moraes et al., 2017, 2018). In brief, bacteria were recovered and grown in LB + Kan. Bacteria were pelleted and lysed. The lysate was used as template for PCR using primers directly flanking the N_18_ barcode. The frequency of each barcode was determined by Illumina sequencing of 20 bases (and is shown for all three *Salmonella* Tn*5* libraries in Supplementary Tables 1A–C). The aggregated abundances for the input and output libraries were statistically analyzed using DESeq2 (Love et al., 2014), and the log_2_-fold changes and FDRs were reported.

### Assessment of the Survival of SGD Mutants on Pistachios

Single-gene deletion mutants of *S*. Typhimurium 14028s were constructed previously (Porwollik et al., 2014). The mutants were grown in 30 ml of LB + Kan in 50-ml conical tubes (VWR Int.) at 37°C for 24 h with shaking. The cultures were pelleted, washed twice in dH_2_O, and used to inoculate 50 g of in-shell pistachios at 4% volume per weight as described above. The inoculated nuts were dried and stored as described above. For *Salmonella* enumerations from the inoculated pistachios immediately after inoculation, after drying and after 30, 65, 85, and 110 days of storage, 2 g of nuts (approximate 2 pistachios) were transferred into 5-ml dH_2_O in a 15-ml conical tube (Becton, Dickinson & Co). The mixtures were vortexed at high speed for 60 s, and appropriate dilutions of the liquid were plated on LB + Kan agar, followed by incubation at 37°C for 24 h. The experiment was conducted in two independent trials with duplicate samples per trial. For mutants that showed significantly higher reduction compared to the wild-type strain, corresponding mutants with the same gene deletion harboring a chloramphenicol resistance gene oriented in the antisense direction (Porwollik et al., 2014) were also tested for survival on pistachios, with enumerations performed on LB + Cm.

### Statistical Analysis

The numbers of colony forming units (CFU) from the survival assays were entered into an Excel 2013 spreadsheet (Microsoft, Redmond, WA, United States), log transformed, exported to SigmaPlot 14.0 (Systat Software Inc., San Jose, CA, United States), and plotted against time to generate survival curves. Comparisons of log reductions between the populations of the wild-type bacteria and the SGD mutants on pistachios at different time points during storage were analyzed using one-way analysis of variance (ANOVA), with Tukey’s test used to compare multiple means (α, 0.05).

## Results and Discussion

### Survival of *Salmonella* Wild-Type Strains and Random Mutant Libraries on Pistachios

**FIGURE 1:**
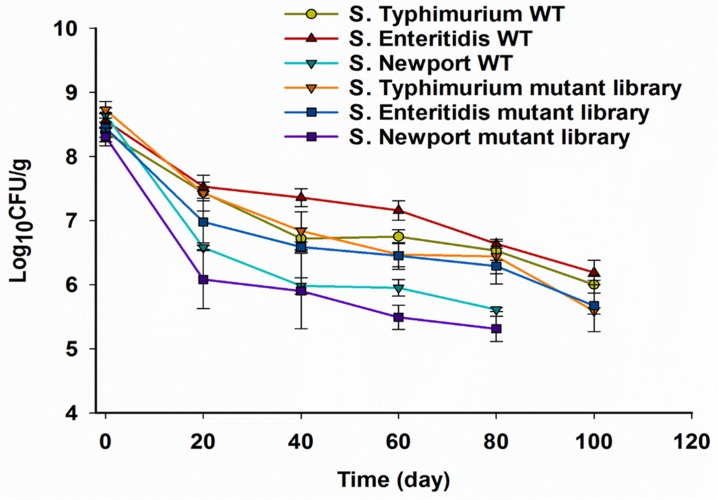
Survival of *Salmonella* wild-type (WT) strains and Tn*5* mutant libraries on in-shell pistachios. Pistachios were inoculated, dried and stored, and populations of *Salmonella* were enumerated as described in section “Materials and Methods” immediately after inoculation, after drying, and periodically during storage. Data represent averages from three independent trials with duplicate samples per trial. Error bars indicate standard deviation (*n* = 6). Enumerations of *S.* Newport C4.2 on pistachios were discontinued when the population was less than 6 log CFU/g.

In the current study, we inoculated pistachios with higher numbers of *Salmonella* than are likely to contaminate LMFs in real life, in order to be able to adequately detect changes during survival and to identify mutants with relevant differences in persistence. The population of *Salmonella* (wild-type strains and mutant libraries) on pistachios immediately after inoculation was approximate 8.5 log CFU/g. The A_w_ of pistachios before inoculation, immediately after inoculation and after drying was 0.25, 0.43, and 0.32, respectively, with no significant changes in A_w_ during storage (data not shown). We noted differences in survival among the three strains that were employed. The population of *S*. Typhimurium 14028s and *S*. Enteritidis P125109 wild-type strains on pistachios was reduced by 1 log CFU/g after drying, with further reductions by 1.5 log CFU/g after storage for 120 days (Figure 1). A higher reduction of 2 log CFU/g was observed in the population of *S*. Newport C4.2 after drying, and the population was below 6 log CFU/g after 30 days of storage (Figure 1).

The observed survival of *S*. Typhimurium 14028s and *S*. Enteritidis P125109 on pistachios in this study corroborates findings from a previous study that reported reductions in the population of *Salmonella* on pistachios by approximate 1 and 1.4 log after drying and during storage at 24°C for 120 days, respectively (Kimber et al., 2012). The noticeably lower survival of *S*. Newport strain C4.2 on pistachios is quite unusual for *Salmonella*. Different *S*. Newport strains have been implicated in LMF-associated *Salmonella* outbreaks and, unlike the Newport strain we used, their desiccation tolerance was found to be comparable to *S.* Typhimurium and Enteritidis (Kirk et al., 2004; Gruzdev et al., 2012b; Centers for Disease Control Prevention [CDC], 2014). However, salmonellosis outbreaks involving strains of serovar Newport tend to be associated with fruit and vegetables (de Moraes et al., 2018). Indeed, functional analysis of the *S*. Newport C4.2 genome revealed unique adaptations to persistence in plants that are not shared by strains of other serotypes, including *S*. Typhimurium 14028s (de Moraes et al., 2018). Therefore, the poor survival of *S*. Newport strain C4.2 in LMFs may reflect trade-offs in this particular strain for adaptations related to plant colonization or perhaps temperate prophage induction under drying stress.

Overall, the survival of wild-type strains was slightly (0.3–0.5 log CFU/g) higher than observed with their respective mutant libraries strains, presumably because some mutants in the libraries are less fit, but this difference was not significant (Figure 1). Both the parental strain and the mutant library of *S*. Newport C4.2 declined markedly relative to the others, with population levels declining to <6 log CFU/g after 30 days of storage (Figure 1). Mutant libraries need to consist of more than 10^6^ distinct mutants in order for the complexity of the library to be maintained and for profiles of mutant fitness to be determined. For this reason, survival assessments of *S*. Newport C4.2 and its mutant library were discontinued after 30 days.

Mutant library populations recovered from the inoculated pistachios immediately after inoculation (T_0_), after drying for 1 day (T_1d_) and after 120 days of storage (T_120d_) were grown in LB + Kan at 37°C for 12 h. This was done to facilitate detachment of strongly adhering cells, resuscitation of injured cells, and extraction of genomic DNA at sufficient concentration for sequencing.

### Tn*5* Insertion Mutants Under Selection in *Salmonella*

**FIGURE 2:**
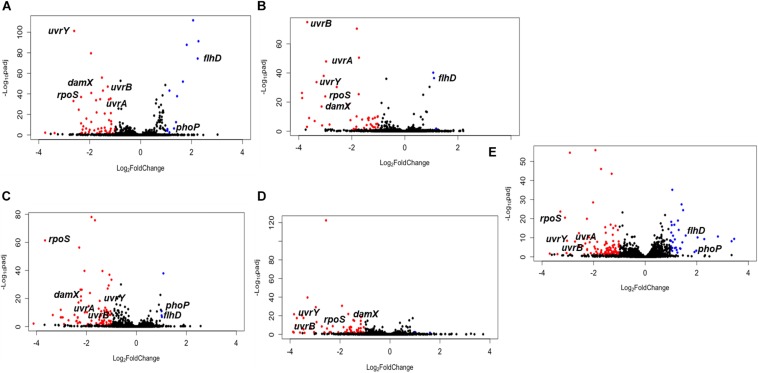
The relative abundance of mutants after selection in pistachios. **(A)** *S.* Enteritidis P125109 mutant library on pistachios after drying for 1 day (T_1d_) relative to immediately after inoculation (T_0_); **(B)** *S.* Enteritidis P125109 mutant library on pistachios at 120 days of storage (T_120d_) relative to T_1d_; **(C)** *S.* Typhimurium 14028s mutant library on pistachios at T_1d_ relative to T_0_; **(D)** *S.* Typhimurium 14028s mutant library on pistachios at T_120d_ relative to T_1d_; **(E)** *S.* Newport C4.2 mutant library on pistachios at T_1d_ relative to T_0_. Red dots indicate significant (*P* < 0.05) reduction by at least two-fold, blue dots indicate significant (*P* < 0.05) increase by at least two-fold, and black dots indicate non-significant and/or less than two-fold change in abundance. Data represent the average of three independent trials and duplicate samples per trial (*n* = 6). Some genes that are consistently under selection and discussed in the text are annotated.

The raw data of barcode counts and the genome positions of Tn*5* insertions used for DESeq2 analysis are reported for all three genomes in the order Typhimurium, Enteritidis, Newport, in Supplementary Table 1. The plots of the relative abundance of Tn*5* insertion sites (TIS) in mutant libraries that were selected on pistachios are shown in Figure 2. TIS with a significant change (*P* < 0.05) in abundance at T_1d_ relative to T_0_, or T_120d_ relative to T_1d_, and with at least a two-fold change (log_2_FC ≥ |1|) were considered to be selected, and thus the corresponding locus was considered to have a putative role in survival. In principle, TIS in mutants impaired for survival would be negatively selected (log_2_FC ≤ −1, *P* < 0.05), as the mutants would have a lower relative abundance on pistachios after drying or storage when compared to the input mutant pools. The corresponding gene would thus have a role in enhancing survival under the experimental conditions. Conversely, for TIS mutants that have higher relative abundance (log_2_FC ≥ 1, *P* < 0.05), the corresponding gene would have a role in reducing survival under the experimental conditions, or might not be required at all. For example, a mutant might be more fit than the wild type if the mutation stops the production of determinants not needed for survival under the tested conditions.

**FIGURE 3:**
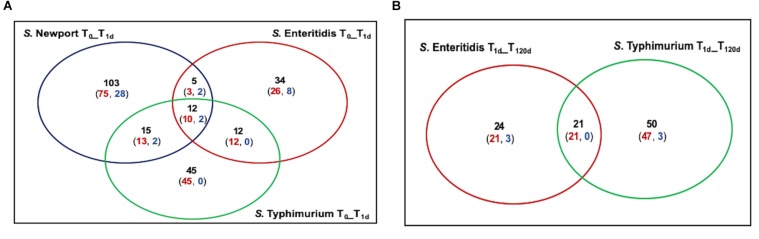
Summary of mutants under selection among the three *Salmonella* serovars. Insertion sites with significant (*P* < 0.05) differences by at least two-fold in relative abundance are shown in panel **(A)** after drying (T_1d_) compared to immediately after inoculation (T_0_), and **(B)** at 120 days of storage (T_120d_) compared to T_1d_. Black font indicate numbers of distinct Tn*5* insertion sites with significant (*P* < 0.05) change by at least two-fold. Red and blue indicate mutants with a reduction and an increase in relative abundance, respectively.

The most extensive selection occurred in the *S*. Newport C4.2 mutant library after drying (T_1d_ versus T_0_) with selection of 135 TIS, of which 101 and 34 were negatively and positively selected, respectively (Figure 3A). This was particularly interesting in light of the above-discussed finding that this strain was markedly more impaired in survival on the dry pistachios than the other two strains (Figure 1). In mutant libraries of *S*. Enteritidis P125109 and *S*. Typhimurium 14028s, 63 and 84 TIS were selected, respectively, with the majority (51 and 80 TIS, respectively) being negatively selected (Figure 3A). Further storage of inoculated pistachios at 25°C for 120 days resulted in further selection of 45 and 71 TIS in the mutant libraries of *S*. Enteritidis P125109 and *S*. Typhimurium 14028s, respectively (Figure 3B). Overall, higher selection occurred during drying than during storage for all mutant libraries, suggesting that drying imposed the highest desiccation-related selection pressure.

*Salmonella enterica* genomes are generally syntenic in over 90% of their genome, with over 95% sequence identity (de Moraes et al., 2018). Concentrating on those part of the genomes shared by at least two of the three strains, the genes disabled by Tn*5* insertions that were significantly changed (*P* < 0.05) in at least two libraries during drying and storage are listed in Table 1. Although the majority of these genes were strain-specific, 53 were significantly changed in at least two mutant libraries while 12 were changed (10 negatively and 2 positively) after drying in all three mutant libraries (Figure 3A). A few of the genes that were selected in two mutant libraries but not in the third one were absent in one specific library (6 in *S*. Newport C4.2 library, 2 in *S*. Enteritidis P125109 library, and 1 in *S*. Typhimurium 14028s library) (Table 1). The genes that were negatively selected in all three mutant libraries during drying included *uvrA*, *uvrB*, *uvrY, rpoS*, *hupA*, *sspA, yifE, rbsR, damX*, and *STM14_2365*, while *phoP* and *flhD* insertions were positively selected in all three libraries (Table 1). Similarly, 21 genes were negatively selected during storage in both the *S*. Enteritidis P125109 and *S*. Typhimurium 14028s mutant libraries (Figure 3B). With the sole exception of the insertion in *hupA*, genes that were negatively selected in all three mutant libraries during drying continued to be further negatively selected during dry storage (Table 1).

**TABLE 1:** Genes under selection in at least two different serovars.

	Log_2_ fold change T_*input*__T_*output*_				
	*S*. Newport T_0____T_1d_	*S*. Enteritidis T_0____T_1d_	*S*. Typhimurium T_0____T_1d_	*S*. Enteritidis T_1d__T_120d_	*S*. Typhimurium T_1d__T_120d_
**DNA repair mechanism**					
STM14_5112 (*uvrA*)*	−**2.56**	−**1.18**	−**1.41**	−**2.97**	−**3.16**
STM14_0926 (*uvrB*)*	−**2.57**	−**1.29**	−**1.48**	−**3.68**	−**4.70**
STM14_4752 (*uvrD*)	NC**	NC	NC	−1.37	−1.33
STM14_2365 (*uvrY*)*	−**2.32**	−**2.61**	−**2.22**	−**3.88**	−**3.52**
STM14_3289 (*recN*)	NC	NC	NC	−1.03	−1.51
STM14_4509 (*recG*)	−1.5	NC	NC	−1.39	−1.25
STM14_4196 (*Dam*)	−3.11	NC	−2.35	NC	NC
STM14_2414 (*Dcm*)	−1.26	NC	−1.2	NC	−1.56
**Osmolarity response**					
STM14_1317 (*mdoG*)	3.45	NC	1.08	NC	NC
STM14_1318 (*mdoH*)	3.35	NC	1.01	NC	NC
STM14_4217 (*ompR*)	NC	NC	NC	−1.81	−3.84
STM14_4216 (*envZ*)	NC	NC	NC	−2.14	−3.73
**Lipopolysaccharide biogenesis**					
STM14_2576 (*rfbP*)	NC	−2.25	−2.95	NC	NC
STM14_2579 (*rfbN*)	ND	−2.00	−3.03	−1.24	−1.18
STM14_2588 (*rfbC*)	NC	−1.37	−2.41	NC	NC
STM14_2590 (*rfbD*)	NC	−1.82	−2.28	NC	NC
STM14_4479 (*rfaL*)	NC	−1.67	−2.25	NC	NC
STM14_4478 (*rfaJ*)	NC	−1.38	−2.35	NC	NC
STM14_4783 (*rfaH*)	−1.55	−2.14	NC	NC	NC
**Transcriptional regulators**					
STM14_1409 (*phoP*)	1.35	1.31	1.01	NC	NC
STM14_3397 (*emrR*)	NC	NC	NC	−1.54	−2.52
STM14_3526 (*rpoS*)*	−**3.29**	−**2.64**	−**3.66**	−**3.33**	−**2.63**
STM14_5011 (*hupA*)	−2.37	−1.24	−1.66	NC	NC
STM14_5249 (*nsrR*)	−1.17	NC	−1.42	NC	−1.65
STM14_2873 (*lrhA*)	−1.03	NC	−1.25	−1.29	−1.28
STM14_5242 (*hfq*)	−2.25	NC	NC	−1.36	−2.42
**Stringent response**					
STM14_4991 (*rpoC*)	−1.29	NC	−1.32	NC	NC
STM14_4506 (*rpoZ*)	NC	−1.19	−1.08	NC	−1.72
STM14_3564 (*relA*)	−1.13	NC	−1.17	NC	−1.28
STM14_4033 (*sspA*)*	−**2.18**	−**1.59**	−**1.86**	−**1.08**	−**3.77**
**Others**					
STM14_0896 (*gpmA*)	−1.97	NC	−2.90	NC	NC
STM14_1106 (*aroA*)	−1.24	−1.02	NC	NC	NC
STM14_1370 (*fabF*)	−1.55	NC	−1.39	NC	NC
STM14_2056 (*rnb*)	−1.94	−1.41	ND***	−1.82	ND
STM14_2341 (*flhD*)	1.01	2.24	1.02	1.10	NC
STM14_3527 (*nlpD*)	NC	−1.75	−2.19	−1.73	NC
STM14_4039 (*yhcB*)	ND	−2.03	−2.07	NC	−3.88
STM14_4694 (*yifE*)*	−**2.02**	−**1.73**	−**2.22**	−**2.05**	−**3.46**
STM14_1755 (*rnfD*)	−1.71	NC	−1.2	NC	NC
STM14_4683 (*rbsR*)*	−**1.55**	−**1.48**	−**1.80**	−**1.13**	−**1.92**
STM14_4682 (*rbsK*)	−1.34	NC	−1.36	NC	−1.47
STM14_3566 (*barA*)	NC	−1.95	−1.08	−3.06	−2.56
STM14_4197 (*damX*)*	−**1.24**	−**1.94**	−**2.24**	−**3.00**	−**1.74**
**Hypothetical proteins**					
*STM14_4207*	2.03	1.66	NC	NC	NC
*STM14_4197.J*	ND	ND	−2.48	ND	−3.85
*STM14_1261*	ND	NC	NC	−2.57	−2.94
STM14_2365.RJ*	−**2.92**	−**2.34**	−**1.91**	−**3.61**	−**3.46**
**Intergenic regions**					
Intergen_1718 (2033472…2033683)	1.05	2.27	NC	1.07	NC
Intergen_2515 (3086724…3086785)	ND	−2.43	−3.03	−3.87	NC
Intergen_3017 (3653422…3653601)	−1.84	NC	−1.98	NC	NC
Intergen_3018 (3654880…3654976)	ND	−2.34	−2.32	−3.69	−3.04
Intergen_3020 (3656644…3657107)	−4.48	NC	−1.3	NC	NC
Intergen_3365 (4120085…4120190)	−1.86	ND	−2.08	ND	2.73

*^∗^Bold type denotes genes impacted in all three libraries. ^∗∗^NC denotes genes or intergens that did not change significantly (Log_2_FC < | 1 | and P < 0.05). ^∗∗∗^ND denotes Tn5 integrations that were absent in the corresponding mutant library and thus not assayed.*

### Genes Putatively Important for Survival of *Salmonella* on Pistachios

Several genes appeared to be important for the survival of *Salmonella* on pistachios. Some of the genes encoded hypothetical proteins while others were implicated in DNA repair, transcriptional regulation, and osmolarity responses. These genes are categorized based on their molecular functions and discussed below.

#### DNA Recombination and Repair Mechanisms

Tn*5* localization in several genes involved in DNA recombination and repair, including *uvrA, uvrB, uvrD*, *recN*, *recG, dam*, and *dcm*, was significantly underrepresented (*P* < 0.05) in the *Salmonella* mutant populations from the dried or stored pistachios, suggesting that the corresponding mutants were impaired for survival on this dry product (Table 1). These observations suggested that *Salmonella* undergoes DNA damage in low-moisture conditions, with survival requiring functional DNA repair systems. UvrA, UvrB and UvrD proteins are central components of the highly conserved nucleotide excision repair (NER) systems in prokaryotes (Kisker et al., 2013). The UvrA-UvrB complex identifies conformational changes induced by DNA lesions, a prerequisite for subsequent steps in NER, and facilitates the recruitment of UvrC, UvrD, DNA polymerase I and DNA ligase for DNA base repair (Truglio et al., 2004; Kisker et al., 2013). On the other hand, RecG, RecN, and UvrD are involved in recombinational repair of DNA damage that often involves double-stranded DNA breaks (Kuzminov, 2011). DNA adenine methyltransferase (Dam) and DNA cytosine methyltransferase (Dcm) are responsible for the majority of methylated DNA bases in *Escherichia coli* and *Salmonella* (Marinus and Løbner-Olesen, 2014). The importance of Dam in DNA mismatch repair, induction of the SOS response, and oxidative stress tolerance has been extensively documented and discussed (Chatti et al., 2012; Marinus and Løbner-Olesen, 2014; Stephenson and Brown, 2016).

Interestingly, *uvrA*, *uvrB* and *uvrY* were selected during both drying and storage, while *dam* and *dcm* were only selected during drying, and *uvrD*, *recN* and *recG* were only selected during storage (Table 1). This differential selection of DNA repair genes suggests that the NER pathway and methylation-directed DNA repair systems were especially important for repair of DNA damage during drying, when viability was also most markedly impacted. The findings agree with data from proteomic analysis of *Salmonella* under desiccation on glass beads which revealed significant abundance of DnaJ and UvrD, proteins involved in DNA repair, compared to cells in the non-desiccated inoculum suspension (Maserati et al., 2018). In addition, a *Salmonella dam* mutant exhibited significantly impaired survival on plastic petri plates (Mandal and Kwon, 2017). On the other hand, none of the DNA recombination and repair genes that appear to be important for survival in the current study were differentially expressed in transcriptome analyses of *Salmonella* under low-moisture conditions (Gruzdev et al., 2012a; Li et al., 2012; Finn et al., 2013b; Maserati et al., 2017). It is worthy of note that these transcriptome studies employed *Salmonella* on desiccated abiotic surfaces such a glass beads, plastics and stainless steel, in contrast to desiccation on pistachios in the current study. Moreover, a gene can be constitutively or transiently expressed and still be important for a phenotype. Thus, some genes that are important for desiccation tolerance of *Salmonella* may not be differentially expressed under desiccation.

The induction of DNA damage in response to desiccation has been reported in other bacteria, including *Deinococcus radiodurans*, *Sinorhizobium meliloti*, *Bradyrhizobium japonicum*, and spores of *Bacillus subtilis* (Dose et al., 1992; Tanaka et al., 2004; Cytryn et al., 2007; Humann et al., 2009). The exact causes of DNA damage under desiccation remain poorly understood, but may include covalent modifications and intracellular accumulation of reactive oxygen species (ROS) (Humann et al., 2009). Moreover, it is not clear if desiccation-induced DNA repair causes adaptive mutations in *Salmonella* that confer higher tolerance to desiccation. For example, exposure of *Listeria monocytogenes* to sublethal acidic pH selected for point mutations in *rpsU*, encoding ribosomal protein S21, that conferred significantly higher tolerance to lethal acidic pH and other stressors (Metselaar et al., 2015). Therefore, the potential of desiccation-induced DNA damage as an adaptive desiccation survival strategy needs to be investigated.

#### Lipopolysaccharide Biogenesis

Several genes involved in LPS biogenesis including *rfbP, rfbN, rfbC, rfbD, rfaL, rfaJ*, and *rfaH* were found to be important for survival of *Salmonella* on pistachios (Table 1). RfbP, RfbN, RfbC, and RfbD are involved in O-antigen synthesis while RfaL, RfaJ, and RfaH mediate LPS core synthesis (Schnaitman and Klena, 1993). Previous studies reported that mutations in LPS biosynthesis genes significantly impaired survival of *Salmonella* on polystyrene petri dishes and formica melamine resin laminate under ambient conditions (Garmiri et al., 2008; Mandal and Kwon, 2017). Furthermore, LPS is a known virulence determinant of *Salmonella* and plays important roles in swarming motility, host invasion and oxidative stress tolerance (Toguchi et al., 2000; Thomsen et al., 2003; Choi and Groisman, 2013; Bogomolnaya et al., 2014).

#### Transcriptional Regulators and Stringent Response

Tn*5* insertions in several transcriptional regulators, including *rpoS*, *lrhA*, *nsrR*, *hupA*, and *emrR*, appeared to result in impaired survival of *Salmonella* on pistachios, by approximate two- to eight-fold during drying, storage, or both (Table 1). Localization in *rpoS* had the greatest impact on survival with approximate eight-fold reduction (log_2_FC = −3) in relative abundance in all libraries during drying, and further reduction by eight-fold during storage (Table 1). The *rpoS* gene encodes the alternative sigma factor RpoS, a global regulator of stress-induced genes, virulence determinants and several metabolic processes in Gram-negative bacteria (Ibañez-Ruiz et al., 2000; Lévi-Meyrueis et al., 2014). The impaired survival on pistachios of *Salmonella* mutants with Tn*5* localization in *rpoS* suggests that certain genes important for survival may be under RpoS control.

Regulation of RpoS intracellular levels is essential for homeostasis in Gram-negative bacteria (Hengge-Aronis, 2002). LrhA, a LysR homolog, is an important regulator of RpoS and other cellular functions such as flagellar synthesis, chemotaxis and NADH-dependent electron transport (Tran et al., 1997; Lehnen et al., 2002; Peterson et al., 2006). Deletion of *lrhA* in *E. coli* resulted in pleiotropic stationary phase defects due to decreased RpoS levels (Gibson and Silhavy, 1999). Mutants with Tn*5* insertions in *lrhA* showed reduced survival during drying and storage in at least two mutant libraries (Table 1). Interestingly, RpoS regulation by LrhA is dependent on the small RNA (sRNA) chaperone Hfq (Peterson et al., 2006), and in the current study *Salmonella* mutants with Tn*5* insertions in *hfq* showed reduced survival during storage (Table 1). These findings suggest that RpoS and its regulation are important for survival of *Salmonella* on LMFs. Interestingly, previous studies did not identify *rpoS, lrhA, hfq* and the other transcriptional regulators detected in the current study as important survival determinants in *Salmonella* under desiccation (Gruzdev et al., 2012a; Li et al., 2012; Finn et al., 2013b; Maserati et al., 2017).

Localization of Tn*5* in *rpoC* and *rpoZ* impaired survival of *Salmonella* on pistachios (Table 1). RpoC and RpoZ make up the β’ and ω subunits of the RNA polymerase and are both involved in the stringent response in *E. coli* (Igarashi et al., 1989; Yamamoto et al., 2018). Mutational studies have shown that the RpoC and RpoZ subunits bind to the alarmone ppGpp which signals the induction of the stringent response in *E. coli*, and the RNA polymerase without these subunits failed to bind to ppGpp (Igarashi et al., 1989; Ross et al., 2013; Zuo et al., 2013). In the current study, insertion of Tn*5* in ppGpp synthase, *relA* and the stringent starvation protein A (*sspA*) impaired survival of *Salmonella* on pistachios (Table 1). These observations suggest that *Salmonella* contaminating LMFs are starved and require stringent response genes for survival.

#### Osmolarity Response

*Salmonella* must be able to detect osmolarity changes in the environment in order to survive under desiccation. The EnvZ/OmpR two-component system is a well-characterized phosphorelay signal transduction system that mediates osmotic stress responses in Gram-negative bacteria (Cai and Inouye, 2002). In the current study, *Salmonella* mutants with Tn*5* insertions in *envZ* and *ompR* were impaired for long-term survival on pistachios (Table 1). Under osmotic stress, EnvZ (inner cytoplasmic membrane-bound histidine kinase) phosphorylates OmpR in the cytoplasm (Cai and Inouye, 2002). Phosphorylated OmpR serves as a pleiotropic transcriptional regulator of outer membrane porin genes such as *ompC* and *ompF* and several other genes, including *ssrA* and *ssrB* that form a two-component regulator of *Salmonella* pathogenicity island 2 (SPI-2) genes and *hilC* which is a known SPI-1 regulator (Lee et al., 2000; Oropeza and Calva, 2009; Cameron and Dorman, 2012). OmpC, OmpF and other membrane porins modulate membrane permeability in Gram-negative bacteria in response to environmental stress (van der Heijden et al., 2016). Therefore, failure to activate the EnvZ/OmpR two-component system in *Salmonella* mutants with Tn*5* insertions in *envZ* and *ompR* was likely responsible for impaired survival on pistachios.

In the *S.* Newport C4.2 and *S*. Typhimurium 14028s mutant libraries, *mdoH* and *mdoG*, encoding osmoregulated periplasmic glucans (Opgs) OpgH and OpgG, respectively (Bohin, 2000), were positively selected during drying but not during storage of inoculated pistachios (Table 1). OpgH and OpgG are believed to catalyze the addition of glucosyl branches to a glucose backbone to form highly branched oligosaccharides that accumulate in the periplasmic space of most Proteobacteria, in response to low-osmolarity environments (Bohin, 2000; Lequette et al., 2004). However, the effect of Opgs on desiccation tolerance has remained elusive. In the current study, inactivation of *mdoH* and *mdoG* appears to be advantageous to survival of *Salmonella* in the LMF model that was investigated.

#### Other Genes and Intergenic Regions

Impaired survival of *Salmonella* on pistachios was associated with localization of Tn*5* in several additional genes (*nlpD, rnfD, rbsR, rbsK, barA, gpmA, aroA, fabF*, and *rnb*) encoding proteins with various known functions as well as several other genes (*yhcB*, *yifE, STM14_4207*, *STM14_4197.J, STM14_1261*, and *STM14_2365.RJ*) encoding putative or hypothetical proteins (Table 1). Of these, only *nlpD* has been previously reported to be putatively involved in *Salmonella* desiccation tolerance (Gruzdev et al., 2012a).

Localization of Tn*5* in six intergenic regions in *Salmonella* was associated with impaired survival on pistachios (Table 2). BLAST analysis suggested that these regions are conserved in diverse serotypes of *Salmonella*. Five of these intergenic regions were flanked by genes in which Tn*5* insertions were also correlated with either impaired (e.g., *rpoS*, *nlpD*, *dam, damX, hdfR*) or enhanced (*flhD*) survival (Table 2). However, mutants with Tn*5* localized in *aroK* and *hofQ*, flanking one of these intergenic regions, were present in the mutant libraries but were not selected in this model system. This may indicate that any phenotype may map inside the intergenic region

**TABLE 2:** Genes flanking the six intergenic regions under selection in *Salmonella* on pistachios.

Intergenic regions	Gene flanking at 5′	Gene flanking at 3′
Intergen_1718	Flagella transcriptional regulator (*flhD*)	Hypothetical protein (*STM14_2343*)
Intergen_2215	Alternative sigma factor (*rpoS*)	Murein hydrolase activator (*nlpD*)
Intergen_3017	DNA adenine methyltransferase (*dam*)	Putative cell division protein (*damX*)
Intergen_3018	Putative cell division protein (*damX*)	3-dehydroquinate synthase (*STM14_4198*)
Intergen_3020	Shikimate kinase gene (*aroK*)	DNA uptake porin gene (*hofQ*)
Intergen_3365	HTH-type transcriptional regulator gene (*hdfR*)	Macrodomain ori organization protein encoding gene (*maoP*).

### Survival of Single Gene Deletion Mutants on Pistachios

Further phenotype evaluation on pistachios was done using individual gene knockout mutants (Porwollik et al., 2014). Genes for this analysis were selected from the Tn*5* data (Table 1) based on a significant (*P* < 0.05) reduction of at least two-fold in relative abundance after drying at T_1d_ versus T_0_ and T_120d_ versus T_1d_ in at least two strains. These mutants included Δ*rbsR*, Δ*damX*, Δ*rfbD*, Δ*lrhA*, Δ*uvrB*, Δ*hupA*, Δ*yhcB*, Δ*ompR*, Δ*barA*, Δ*uvrY*, Δ*yifE*, and Δ*sspA*, and mutants in six of these genes (*uvrB*, *uvrY*, *sspA*, *damX*, *rbsR*, and *yifE*) were negatively selected in all three libraries at T_1d_ versus T_0_ (Table 1).

**FIGURE 4:**
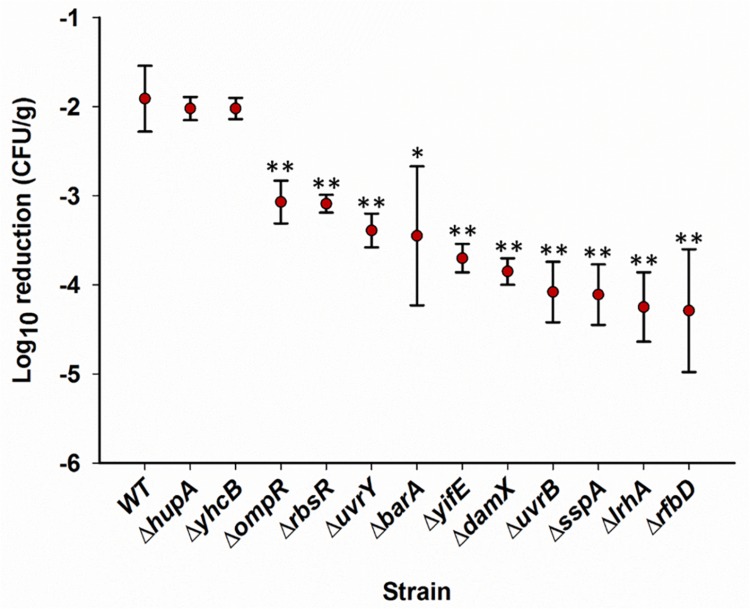
Survival of individual single-gene deletion mutants on in-shell pistachios. Pistachios were inoculated with single strains, dried and stored. Populations of *Salmonella* were enumerated as log_10_CFU/g, as described in “Materials and Methods”, immediately after inoculation, after drying, and periodically during storage. The difference between the population immediately after inoculation and at 110 days of storage is plotted for each mutant. Data represent averages from two independent trials with duplicate samples processed per trial. Error bars indicate standard deviation (*n* = 4). ^∗^*P* < 0.05, ^∗∗^*P* < 0.005, based on two tailed Student’s *t*-test. Supplementary Table 2 contains data for all time points.

The wild-type *Salmonella* and the SGD mutants inoculated on pistachios ranged from 8.23 to 8.79 log CFU/g, with reductions by approximate 1 log CFU/g after drying for 1 day. After storage of inoculated pistachios at 25°C, most of the individual single gene deletion mutants, chosen for further study of their potential positive role of the deleted gene in survival, showed impairment in survival at all time points (Supplementary Table 2). At 110 days, 10 of the 12 SGD mutants showed significantly (*P* < 0.05) impaired survival with population reductions exceeding those of the wild-type strain by more than 100-fold for mutants Δ*rfbD*, Δ*lrhA*, Δ*uvrB*, and Δ*sspA* (Figure 4). In contrast, survival of two SGD mutants, Δ*hupA* and Δ*yhcB*, was similar to that of the parental strain throughout the 110 days (Figure 4). This is in contrast to the negative selection of Tn5 mutations in these genes when in competition with other *Salmonella* in a library. One possible reason for this difference in phenotype may be that these mutants compete less effectively when bacteria with a wild-type phenotype are present but are not impaired when surviving alone on pistachios. Alternatively, a Tn5 insertion may alter the function of a gene rather than eliminate it, or may have different polar effects on a neighboring CDS, compared to a whole gene deletion. Overall, the phenotypes of 10 of 12 SGD mutants tested for a positive role in survival corroborated the findings from the transposon sequencing analysis of the mutant libraries. These experiments were performed using gene knockouts with a kanamycin resistant cassette (Porwollik et al., 2014). Similar data were obtained with the chloramphenicol-resistant derivatives of these SGD mutants (data not shown).

Most of the genes identified by mutagenesis to be important for survival in the current study were not identified in previous studies that used differentially expressed RNAs or proteins as an assay to investigate the molecular basis of survival of *Salmonella* (Gruzdev et al., 2012a; Li et al., 2012; Finn et al., 2013b; Maserati et al., 2017). One explanation for some of these differences may be that we have measured the effect of mutation on survival directly, whereas the levels of RNA or protein do not necessarily correlate with the importance of the respective gene for survival (Price et al., 2013). This is supported by the finding that deletion of some genes that were previously reported to be differentially expressed in *Salmonella* under low-moisture conditions did not impact survival (Gruzdev et al., 2012a; Finn et al., 2013b). Another explanation may be that the previous studies primarily tested survival of *Salmonella* on abiotic surfaces and for shorter period of time. Thus, *Salmonella* may deploy different desiccation survival strategies depending on other factors in the environment.

## Conclusion

In the current study, we have integrated transposon mutagenesis and sequence analysis to identify novel determinants important for survival of *Salmonella* on pistachios, employed as a model of low-moisture food (LMF). Our findings suggest that DNA recombination and repair, osmolarity regulation, lipopolysaccharide biogenesis, stringent response, and stress-induced sigma factors are some of the mechanisms employed by *Salmonella* to survive on LMFs. Elucidation of the molecular basis of the survival of *Salmonella* on LMFs may aid the development of improved detection and inactivation strategies, thereby reducing the food safety burden associated with contamination of LMFs.

## Data Availability Statement

The datasets generated for this study can be found in the Supplementary Tables 1 and 2.

## Author Contributions

MM, VJ, and SK conceived the study. VJ, MM, SP, and WC performed the experiments and analyzed the data. VJ drafted the manuscript. SK, MM, and SP revised the manuscript. JF, MM, and SK secured the funding.

## Conflict of Interest

The authors declare that the research was conducted in the absence of any commercial or financial relationships that could be construed as a potential conflict of interest.

## References

[B1] Beuchat L. R. Komitopoulou E. Beckers H. Betts R. P. Bourdichon F. Fanning S. (2013). Low-water activity foods: increased concern as vehicles of foodborne pathogens. *J. Food Prot.* 76 150–172. 10.4315/0362-028X.JFP-12-211 23317872

[B2] Beuchat L. R. Mann D. A. (2014). Survival of *Salmonella* on dried fruits and in aqueous dried fruit homogenates as affected by temperature. *J. Food Prot.* 77 1102–1109. 10.4315/0362-028X.JFP-13-549 24988015

[B3] Bogomolnaya L. M. Aldrich L. Ragoza Y. Talamantes M. Andrews K. D. McClelland M. (2014). Identification of novel factors involved in modulating motility of *Salmonella enterica* serotype typhimurium. *PloS One* 9:e111513. 10.1371/journal.pone.0111513 25369209 PMC4219756

[B4] Bohin J. P. (2000). Osmoregulated periplasmic glucans in *Proteobacteria*. *FEMS Microbiol. Lett.* 186 11–19. 10779706 10.1111/j.1574-6968.2000.tb09075.x

[B5] Cai S. J. Inouye M. (2002). EnvZ-OmpR interaction and osmoregulation in *Escherichia coli*. *J. Biol. Chem.* 277 24155–24161. 11973328 10.1074/jbc.M110715200

[B6] Cameron A. D. S. Dorman C. J. (2012). A fundamental regulatory mechanism operating through OmpR and DNA topology controls expression of *Salmonella* pathogenicity islands SPI-1 and SPI-2. *PLoS Genet.* 8:e1002615. 10.1371/journal.pgen.1002615 22457642 PMC3310775

[B7] Centers for Disease Control Prevention [CDC] (2009). Multistate outbreak of *Salmonella* infections associated with peanut butter and peanut butter-containing products—United States, 2008–2009. *Morb. Mortal. Wkly. Rep.* 58 85–90. 19194370

[B8] Centers for Disease Control Prevention [CDC] (2014). *Multistate Outbreak of Salmonella Infections Linked to Organic Sprouted Chia Powder.* Available at https://www.cdc.gov/salmonella/newport-05-14/index.html. (accessed on September 24, 2019).

[B9] Centers for Disease Control Prevention [CDC] (2016). *Multistate Outbreak of Salmonella Montevideo and Salmonella Senftenberg Infections Linked to Wonderful Pistachios (Final Update).* Available at https://www.cdc.gov/salmonella/montevideo-03-16/index.html (accessed on October 19, 2019)^∗^

[B10] Centers for Disease Control Prevention [CDC] (2019). *Reports of Selected Salmonella Outbreak Investigations.* Available at https://www.cdc.gov/salmonella/outbreaks.html. (accessed on October 23, 2019)^∗^

[B11] Chatti A. Messaoudi N. Mihoub M. Landoulsi A. (2012). Effects of hydrogen peroxide on the motility, catalase and superoxide dismutase of *dam* and/or *seqA* mutant of *Salmonella* Typhimurium. *World J. Microbiol. Biotechnol.* 28 129–133. 10.1007/s11274-011-0801-8 22806788

[B12] Choi J. Groisman E. A. (2013). The lipopolysaccharide modification regulator PmrA limits *Salmonella* virulence by repressing the type three-secretion [sic] system Spi/Ssa. *Proc. Natl. Acad. Sci. U.S.A.* 110 9499–9504. 10.1073/pnas.1303420110 23690578 PMC3677452

[B13] Crucello A. Furtado M. M. Chaves M. D. R. Sant’Ana A. S. (2019). Transcriptome sequencing reveals genes and adaptation pathways in *Salmonella* Typhimurium inoculated in four low water activity foods. *Food Microbiol.* 82 426–435. 10.1016/j.fm.2019.03.016 31027802

[B14] Csonka L. N. Hanson A. D. (1991). Prokaryotic osmoregulation: genetics and physiology. *Annu. Rev. Microbiol.* 45 569–606. 1741624 10.1146/annurev.mi.45.100191.003033

[B15] Cytryn E. J. Sangurdekar D. P. Streeter J. G. Franck W. L. Chang W. S. Stacey G. (2007). Transcriptional and physiological responses of *Bradyrhizobium japonicum* to desiccation-induced stress. *J. Bacteriol.* 189 6751–6762. 17660288 10.1128/JB.00533-07 PMC2045231

[B16] de Moraes M. H. Becerra S. E. Salas G. I. Desai P. Chu W. Porwollik S. (2018). Genome-wide comparative functional analyses reveal adaptations of Salmonella sv. Newport to a plant colonization lifestyle. *Front. Microbiol.* 9:877. 10.3389/fmicb.2018.00877 29867794 PMC5968271

[B17] de Moraes M. H. Desai P. Porwollik S. Canals R. Perez D. R. Chu W. (2017). *Salmonella* persistence in tomatoes requires a distinct set of metabolic functions identified by transposon insertion sequencing. *Appl. Environ. Microbiol.* 83:e03028-16. 10.1128/AEM.03028-16 28039131 PMC5311394

[B18] Dose K. Bieger-Dose A. Labusch M. Gill M. (1992). Survival in extreme dryness and DNA-single-strand breaks. *Adv. Space Res.* 12 221–229. 11538142 10.1016/0273-1177(92)90176-x

[B19] Farakos S. M. S. Frank J. F. (2014). “Challenges in the control of foodborne pathogens in low-water activity foods and spices,” in *The Microbiological Safety of Low Water Activity Foods and Spices*, eds Gurtler J. B. Doyle M. P. Kornacki J. L. (New York, NY: Springer), 15–34.

[B20] Finn S. Condell O. McClure P. Amézquita A. Fanning S. (2013a). Mechanisms of survival, responses, and sources of *Salmonella* in low-moisture environments. *Front. Microbiol.* 4:331 10.3389/fmicb.2013.00331 PMC3827549 24294212

[B21] Finn S. Händler K. Condell O. Colgan A. Cooney S. McClure P. (2013b). ProP is required for the survival of desiccated *Salmonella enterica* serovar Typhimurium cells on a stainless steel surface. *Appl. Environ. Microbiol.* 79 4376–4384. 10.1128/AEM.00515-13 23666329 PMC3697505

[B22] Garmiri P. Coles K. E. Humphrey T. J. Cogan T. A. (2008). Role of outer membrane lipopolysaccharides in the protection of *Salmonella enterica* serovar Typhimurium from desiccation damage. *FEMS Microbiol. Lett.* 281 155–159. 10.1111/j.1574-6968.2008.01093.x 18312578

[B23] Gibson K. E. Silhavy T. J. (1999). The LysR homolog LrhA promotes RpoS degradation by modulating activity of the response regulator SprE. *J. Bacteriol.* 181 563–571. 9882671 10.1128/jb.181.2.563-571.1999 PMC93411

[B24] Gruzdev N. McClelland M. Porwollik S. Ofaim S. Pinto R. Saldinger-Sela S. (2012a). Global transcriptional analysis of dehydrated *Salmonella enterica* serovar Typhimurium. *Appl. Environ. Microbiol.* 78 7866–7875. 10.1128/AEM.01822-12 22941081 PMC3485933

[B25] Gruzdev N. Pinto R. Sela S. S. (2012b). Persistence of *Salmonella enterica* during dehydration and subsequent cold storage. *Food Microbiol.* 32 415–422. 10.1016/j.fm.2012.08.003 22986208

[B26] Gruzdev N. Pinto R. Sela S. (2011). Effect of desiccation on tolerance of *Salmonella enterica* to multiple stresses. *Appl. Environ. Microbiol.* 77 1667–1673. 10.1128/AEM.02156-10 21216905 PMC3067256

[B27] Hengge-Aronis R. (2002). Signal transduction and regulatory mechanisms involved in control of the sigma(S) (RpoS) subunit of RNA polymerase. *Microbiol. Mol. Biol. Rev.* 66 373–395. 12208995 10.1128/MMBR.66.3.373-395.2002 PMC120795

[B28] Hokunan H. Koyama K. Hasegawa M. Kawamura S. Koseki S. (2016). Survival kinetics of *Salmonella enterica* and enterohemorrhagic *Escherichia coli* on a plastic surface at low relative humidity and on low–water activity foods. *J. Food Prot.* 79 1680–1692. 10.4315/0362-028X.JFP-16-081 28221855

[B29] Humann J. L. Ziemkiewicz H. T. Yurgel S. N. Kahn M. L. (2009). Regulatory and DNA repair genes contribute to the desiccation resistance of *Sinorhizobium meliloti* Rm1021. *Appl. Environ. Microbiol.* 75 446–453. 10.1128/AEM.02207-08 19028909 PMC2620701

[B30] Ibañez-Ruiz M. Robbe-Saule V. Hermant D. Labrude S. Norel F. (2000). Identification of RpoS (sigma S)-regulated genes in *Salmonella enterica* Serovar Typhimurium. *J. Bacteriol.* 182 5749–5756. 11004173 10.1128/jb.182.20.5749-5756.2000 PMC94696

[B31] Igarashi K. Fujita N. Ishihama A. (1989). Promoter selectivity of *Escherichia coli* RNA polymerase: omega factor is responsible for the ppGpp sensitivity. *Nucleic Acids Res.* 17 8755–8765. 2685748 10.1093/nar/17.21.8755 PMC335041

[B32] Isaacs S. Aramini J. Ciebin B. Farrar J. A. Ahmed R. Middleton D. (2005). An international outbreak of salmonellosis associated with raw almonds contaminated with a rare phage type of *Salmonella* Enteritidis. *J. Food Prot.* 68 191–198. 15690826 10.4315/0362-028x-68.1.191

[B33] Jarvik T. Smillie C. Groisman E. A. Ochman H. (2010). Short-term signatures of evolutionary change in the *Salmonella enterica* Serovar Typhimurium 14028 genome. *J. Bacteriol.* 192 560–567. 10.1128/JB.01233-09 19897643 PMC2805332

[B34] Kimber M. A. Kaur H. Wang L. Danyluk M. D. Harris L. J. (2012). Survival of Salmonella, *Escherichia coli* O157:H7, and Listeria monocytogenes on inoculated almonds and pistachios stored at -19, 4, and 24°C. *J. Food Prot.* 75 1394–1403. 22856562 10.4315/0362-028X.JFP-12-023

[B35] Kirk M. D. Little C. L. Lem M. Fyfe M. Genobile D. Tan A. (2004). An outbreak due to peanuts in their shell caused by *Salmonella enterica* serotypes Stanley and Newport- sharing molecular information to solve international outbreaks. *Epidemiol. Infect.* 132 571–577. 15310157 10.1017/s095026880400216x PMC2870136

[B36] Kisker C. Kuper J. Van Houten B. (2013). Prokaryotic nucleotide excision repair. *Cold Spring Harb. Perspect. Biol.* 5:a012591. 10.1101/cshperspect.a012591 23457260 PMC3578354

[B37] Kuzminov A. (2011). Homologous recombination-experimental systems, analysis, and significance. *EcoSal Plus* 4:10.1128/ecosallus.7.2.6. 10.1128/ecosalplus.7.2.6 PMC4190071 26442506

[B38] Lee A. K. Detweiler C. S. Falkow S. (2000). OmpR regulates the two-component system SsrA-SsrB in *Salmonella* pathogenicity island 2. *J. Bacteriol.* 182 771–781. 10633113 10.1128/jb.182.3.771-781.2000 PMC94342

[B39] Lehnen D. Blumer C. Polen T. Wackwitz B. Wendisch V. F. Unden G. (2002). LrhA as a new transcriptional key regulator of flagella, motility and chemotaxis genes in *Escherichia coli*. *Mol. Microbiol.* 45 521–532. 12123461 10.1046/j.1365-2958.2002.03032.x

[B40] Lequette Y. Odberg-Ferragut C. Bohin J. P. Lacroix J. M. (2004). Identification of *mdoD* and *mdoG* paralog which encodes a twin-arginine-dependent periplasmic protein that controls osmoregulated periplasmic glucan backbone structures. *J. Bacteriol.* 186 3695–3702. 15175282 10.1128/JB.186.12.3695-3702.2004 PMC419940

[B41] Lévi-Meyrueis C. Monteil V. Sismeiro O. Dillies M.-A. Monot M. Jagla B. (2014). Expanding the RpoS/σS-Network by RNA sequencing and identification of σS-controlled small RNAs in *Salmonella*. *PLoS One* 9:e96918. 10.1371/journal.pone.0096918 24810289 PMC4014581

[B42] Li H. Bhaskara A. Megalis C. Tortorello M. L. (2012). Transcriptomic analysis of *Salmonella* desiccation resistance. *Foodborne Pathogens Dis.* 9 1143–1151. 10.1089/fpd.2012.1254 23237410

[B43] Love M. I. Huber W. Anders S. (2014). Moderated estimation of fold change and dispersion for RNA-seq data with DESeq2. *Genome Biol.* 15:550. 25516281 10.1186/s13059-014-0550-8 PMC4302049

[B44] Mandal R. K. Kwon Y. M. (2017). Global Screening of *Salmonella enterica* Serovar Typhimurium genes for desiccation survival. *Front. Microbiol.* 8:1723. 10.3389/fmicb.2017.01723 28943871 PMC5596212

[B45] Marinus M. G. Løbner-Olesen A. (2014). DNA methylation. *EcoSal Plus* 6:10.1128. 10.1128/ecosalplus.ESP-0003-2013 PMC4231299 26442938

[B46] Maserati A. Fink R. C. Lourenco A. Julius M. L. Diez-Gonzalez F. (2017). General response of *Salmonella enterica* serovar Typhimurium to desiccation: a new role for the virulence factors *sopD* and *sseD* in survival. *PLoS One* 12:e0187692. 10.1371/journal.pone.0187692 29117268 PMC5678696

[B47] Maserati A. Lourenco A. Diez-Gonzalez F. Fink R. C. (2018). iTRAQ-Based global proteomic analysis of *Salmonella enterica* Serovar Typhimurium in response to desiccation, low water activity, and thermal treatment. *Appl. Environ. Microbiol.* 84:e0393-18. 10.1128/AEM.00393-18 29959250 PMC6121987

[B48] Mattick K. L. Jørgensen F. Legan J. D. Cole M. B. Porter J. Lappin-Scott H. M. (2000). Survival and filamentation of *Salmonella enterica* serovar Enteritidis PT4 and *Salmonella enterica* serovar Typhimurium DT104 at low water activity. *Appl. Environ. Microbiol.* 66 1274–1279. 10742199 10.1128/aem.66.4.1274-1279.2000 PMC91980

[B49] Metselaar K. I. den Besten H. M. Boekhorst J. van Hijum S. A. Zwietering M. H. Abee T. (2015). Diversity of acid stress resistant variants of *Listeria monocytogenes* and the potential role of ribosomal protein S21 encoded by *rpsU*. *Front. Microbiol.* 6:422. 10.3389/fmicb.2015.00422 26005439 PMC4424878

[B50] Oropeza R. Calva E. (2009). The cysteine 354 and 277 residues of *Salmonella enterica* serovar Typhi EnvZ are determinants of autophosphorylation and OmpR phosphorylation. *FEMS Microbiol. Lett.* 292 282–290. 10.1111/j.1574-6968.2009.01502.x 19187206

[B51] Peterson C. N. Carabetta V. J. Chowdhury T. Silhavy T. J. (2006). LrhA regulates rpoS translation in response to the Rcs phosphorelay system in *Escherichia coli*. *J. Bacteriol.* 188 3175–3181. 16621809 10.1128/JB.188.9.3175-3181.2006 PMC1447435

[B52] Porwollik S. Santiviago C. A. Cheng P. Long F. Desai P. Fredlund J. (2014). Defined single-gene and multi-gene deletion mutant collections in *Salmonella enterica* sv Typhimurium. *PLoS One.* 9:e99820. 10.1371/journal.pone.0099820 25007190 PMC4089911

[B53] Price M. N. Deutschbauer A. M. Skerker J. M. Wetmore K. M. Ruths T. Mar J. S. (2013). Indirect and suboptimal control of gene expression is widespread in bacteria. *Mol. Syst. Biol.* 9:660. 10.1038/msb.2013.16 23591776 PMC3658271

[B54] Ross W. Vrentas C. E. Sanchez-Vazquez P. Gaal T. Gourse R. L. (2013). The magic spot: a ppGpp binding site on *E. coli* RNA polymerase responsible for regulation of transcription initiation. *Mol. Cell* 50 420–429. 10.1016/j.molcel.2013.03.021 23623682 PMC3654024

[B55] Schnaitman C. A. Klena J. D. (1993). Genetics of lipopolysaccharide biosynthesis in enteric bacteria. *Microbiol. Rev.* 57 655–682. 7504166 10.1128/mr.57.3.655-682.1993 PMC372930

[B56] Smith D. F. Hildebrandt I. M. Casulli K. E. Dolan K. D. Marks B. P. (2016). Modeling the effect of temperature and water activity on the thermal resistance of *Salmonella* Enteritidis PT 30 in wheat flour. *J. Food Prot.* 79 2058–2065. 10.4315/0362-028X.JFP-16-155 28221962

[B57] Stephenson S. A. Brown P. D. (2016). Epigenetic influence of Dam methylation on gene expression and attachment in uropathogenic *Escherichia coli*. *Front. Public Health* 4:131. 10.3389/fpubh.2016.00131 27446897 PMC4921776

[B58] Tanaka M. Earl A. M. Howell H. A. Park M. J. Eisen J. A. Peterson S. N. (2004). Analysis of *Deinococcus radiodurans’*s transcriptional response to ionizing radiation and desiccation reveals novel proteins that contribute to extreme radioresistance. *Genetics* 168 21–33. 15454524 10.1534/genetics.104.029249 PMC1448114

[B59] Thomsen L. E. Chadfield M. S. Bispham J. Wallis T. S. Olsen J. E. Ingmer H. (2003). Reduced amounts of LPS affect both stress tolerance and virulence of *Salmonella enterica* serovar Dublin. *FEMS Microbiol. Lett.* 228 225–231. 14638428 10.1016/S0378-1097(03)00762-6

[B60] Toguchi A. Siano M. Burkart M. Harshey R. M. (2000). Genetics of swarming motility in *Salmonella enterica* serovar typhimurium: critical role for lipopolysaccharide. *J. Bacteriol.* 182 6308–6321. 11053374 10.1128/jb.182.22.6308-6321.2000 PMC94776

[B61] Toro M. Retamal P. Ayers S. Barreto M. Allard M. Brown E. W. (2016). Whole-genome sequeincing analysis of *Salmonella enterica* serovar Enteritidis isolates in Chile provides insights into possible transmission between gulls, poultry, and humans. *Appl. Environ. Microbiol.* 82 6223–6232. 27520817 10.1128/AEM.01760-16 PMC5068155

[B62] Tran Q. H. Bongaerts J. Vlad D. Unden G. (1997). Requirement for the proton-pumping NADH dehydrogenase I of *Escherichia coli* in respiration of NADH to fumarate and its bioenergetic implications. *Eur. J. Biochem.* 244 155–160. 9063459 10.1111/j.1432-1033.1997.00155.x

[B63] Truglio J. J. Croteau D. L. Skorvaga M. DellaVecchia M. J. Theis K. Mandavilli B. S. (2004). Interactions between UvrA and UvrB: the role of UvrB’s domain 2 in nucleotide excision repair. *EMBO J.* 23 2498–2509. 15192705 10.1038/sj.emboj.7600263 PMC449773

[B64] van der Heijden J. Reynolds L. A. Deng W. Mills A. Scholz R. Imami K. (2016). *Salmonella* rapidly regulates membrane permeability to survive oxidative stress. *MBio* 7 1238–1216. 10.1128/mBio.01238-16 27507830 PMC4992977

[B65] White A. P. Gibson D. L. Kim W. Kay W. W. Surette M. G. (2006). Thin aggregative fimbriae and cellulose enhance long-term survival and persistence of *Salmonella*. *J. Bacteriol.* 188 3219–3227. 16621814 10.1128/JB.188.9.3219-3227.2006 PMC1447457

[B66] Yamamoto K. Yamanaka Y. Shimada T. Sarkar P. Yoshida M. Bhardwaj N. (2018). Altered distribution of RNA polymerase lacking the omega subunit within the prophages along the *Escherichia coli* K-12 genome. *MSystems* 3:e0172-17. 10.1128/mSystems.00172-17 29468196 PMC5811629

[B67] Zuo Y. Wang Y. Steitz T. A. (2013). The mechanism of *E. coli* RNA polymerase regulation by ppGpp is suggested by the structure of their complex. *Mol. Cell* 50 430–436. 23623685 10.1016/j.molcel.2013.03.020 PMC3677725

